# David Chidester: Wild Religion: Tracking the Sacred in South Africa: University of California Press, Berkeley, 2012, p. 280, $28.95

**DOI:** 10.1007/s13644-013-0141-2

**Published:** 2013-09-25

**Authors:** Hans Jurgens Hendriks

**Affiliations:** Network for African Congregational Theology (NetACT), Stellenbosch University, Stellenbosch, South Africa

David Chidester’s *Wild Religion* focuses on religious diversity and South Africa “from the advent of democracy in 1994 to the euphoria of the Football World Cup in 2010” (viii). It is not a conventionally defined description of particular faith communities or sacred rituals but rather an attempt to describe in a well-informed story-telling anecdotal way how ritual and the sacred are used (and misused) “for negotiating a human identity” in times of transition. Thus it is “wild” religion, a reaction to a new and “wild” world that throws both punches and surprises. The “sacred” is then used to either vent frustration and anger—thus disruptive of social order, or it can be used in a way to deal with it, to pacify and interpret it, to recreate social order. It is “a hybrid of order and chaos” (4).

*Wild Religion* is a sociological study of religion in an extremely interesting time and place written in an original, scholarly and entertaining way focusing on a truly diverse and multicultural society in transition. However, it discusses the phenomena with examples from all over the world and from various times in history. It never gets dull but it often made me wonder if all of this is really an accurate and a just rendering of reality. One can prove your point that the religious scenario is actually constant chaos by simply telling and quoting enough (well documented!) stories. It truly is a bit wild, like the hair raising stories about hair (5–10)! But I guess that is the point that the author wants to make, it is a messy chaos even though one can find a basic logic that helps one to understand the constant fluctuations.

What exactly this basic logic is becomes clear and is well articulated in the final chapter. After critiquing systematic descriptions of the religious system of the Amazulu and the highly respected and often quoted work of John Mbiti, the author concludes that such “utopian” religious systems simply never existed (192). He continues (192):

Neither a stable and enduring system nor a perennial mentality that persisted unchanged from time immemorial, African religion is an open set of resources and strategies for sacralising.

The word “transact” and “transactions” are often used. They refer to the coping mechanism that people and people groups use to deal with changing realities. The setting of the changing reality is usually illustrated from the context of colonialism as well as the modern era. Religion, in utterly original ways (called “wild”), used the sacred to cope with identity changes and used and misused the processes to the advantage of persons or peoples, often politically, economically and socially. On page 196, after some interesting illustrations, the author once again concludes:

…these cases utterly defy any construction of African traditional religion as a stable system or a distinctive mentality. They demonstrate the relational character of indigenous religion. The transactions suggest the variety of ways in which indigenous religion is not preserved in splendid isolation from time immemorial but always entangled in a changing world.

The different chapters of the book document the evidence that leads to these conclusions. Chapter two, for instance, maps the morphology of the sacred by looking at the symbolic in the history of Cape Town being the mother city of South Africa. In a truly fascinating way he outlines his case which is a particular understanding of religion (44):

I would like to propose in conclusion that the term *religion* can be recast to designate a category of human activity that comprises not only beliefs and practices, whether in relation to transcendent forces, sacred objects or ultimate concerns, but also resources and strategies – the resources that are appropriated and the strategies that are deployed – within an urban political economy of the sacred.

His interpretation of this definition of religion is then very well illustrated by interpreting the recent history of urban gangs in Cape Town with regard to the role that the sacred played. The rest of the chapters deal with violence, fundamentalism, heritage, spirituality, and the quest for purity and power as well as an enjoyable chapter of the influence of the soccer World Cup on the country and on religion in particular.

Another very important conclusion one finds on page 198. He calls religion in South Africa “wild” because of its preoccupation with modernity, trying to cope with “what’s next.” “Christianity and indigenous religion have been interwoven in South Africa.” I think this is an important conclusion and applicable to most of sub-Saharan Africa.

Whether the hope that the book promised for South Africa is indeed real hope is open to be questioned for the prophecy with which it ends is that religion will stay wild.

